# Morphometric Characteristics of the Spermatozoa of Blue Fox (*Alopex lagopus*) and Silver Fox (*Vulpes vulpes*)

**DOI:** 10.3390/ani10101927

**Published:** 2020-10-20

**Authors:** Katarzyna Andraszek, Dorota Banaszewska, Olga Szeleszczuk, Marta Kuchta-Gładysz, Anna Grzesiakowska

**Affiliations:** 1 Institute of Animal Science and Fisheries, Faculty of Agrobioengineering and Animal Husbandry, Siedlce University of Natural Sciences and Humanities, Prusa 14 Str, 08–110 Siedlce, Poland; dorota.banaszewska@uph.edu.pl; 2 Department of Animal Reproduction, Anatomy and Genomics, Faculty of Animal Sciences, University of Agriculture in Krakow, Mickiewicza 24/28 Str, 30–059 Kraków, Poland; rzszeles@cyf-kr.edu.pl (O.S.); marta.kuchta-gladysz@urk.edu.pl (M.K.-G.); anna.grzesiakowska@student.urk.edu.pl (A.G.)

**Keywords:** fox, sperm morphometry, silver nitrate, Tygerberg criteria

## Abstract

**Simple Summary:**

The present study describes a detailed morphometric analysis of the sperm of the blue fox (*Alopex lagopus*) and silver fox (*Vulpes vulpes*), together with determination of the shape indices of the sperm head. Staining with silver nitrate enables precise identification of the acrosome and reveals structural details of the sperm tail, so that they can be accurately measured. Statistically significant differences were found for most of the morphometric parameters of the two fox species. The blue fox sperm were generally larger, but the acrosome area and coverage were greater in the silver fox. There are no clear recommendations regarding sperm staining techniques for foxes, and no reference values for morphometric parameters of the sperm of foxes or for canines in general. Staining with silver nitrate for evaluation of the morphometry of fox sperm can be used as an independent technique or an auxiliary technique in routine analysis of canine semen.

**Abstract:**

The results presented in this study are the first such extensive characterization of the sperm morphometry of the blue fox (*Alopex lagopus*) and silver fox (*Vulpes vulpes*), as representatives of the family Canidae. Canine spermatozoa, especially the sperm of farmed foxes, are not often described in studies on reproduction. The aim of the study was a detailed comparison of the morphometric dimensions and shape of the sperm of two fox species: silver fox and blue fox. Semen collected from 10 silver foxes and 10 blue foxes was used for the study. The specimens were stained with silver nitrate. Measurements were performed of the length, width, perimeter, and area of the head; the area of the acrosome and its coverage; the length of the midpiece and its coverage; the length of the tail; and the length of the end piece of the tail. In addition, four head shape indices were calculated: ellipticity, elongation, roughness and regularity. The following values for the morphometric parameters and shape indices were obtained for blue fox and silver fox, respectively: head length—6.72 µm and 6.33 µm; head width—4.54.µm and 4.21 µm; head perimeter—18.11 µm and 17.37 µm; head area—21.94 µm^2^ and 21.11 µm^2^; acrosome area—11.50 µm^2^ and 10.92 µm^2^; midpiece length—12.85 µm and 12.79 µm; tail end piece length—3.44 µm and 3.28 µm; tail length—65.23 µm and 65.09 µm; acrosome coverage—52.43% and 52.83%; midpiece coverage—19.71% and 19.65%; sperm length—71.95 µm and 71.42 µm; ellipticity—1.49 and 1.52; elongation—0.19 and 0.20; roughness—0.84 and 1.88; regularity—1.09 and 0.99. The significance of differences between species was verified by Tukey’s test at *p* ≤ 0.05. Statistically significant differences between species were found for the following parameters: head length, width, perimeter and area; acrosome area; tail, end piece, and total sperm length; roughness and regularity. The differences in the size and shape of sperm can be used to establish reference patterns for fox sperm enabling more accurate species identification.

## 1. Introduction

Fox farming is an important branch of production in many countries of Northern Europe, Asia, and Africa [1]. Techniques for managing fox reproduction are relatively simple [1,2]. However, knowledge about general reproductive function is still limited [3]. Examination of sperm morphology remains an important step in assessing the fertility of individuals and their response to cryopreservation [4,5]. The spermatozoa of foxes are homomorphous, with a relatively low level of morphological abnormalities (on average about 10%) [6]. Jalkanen [7] indicate a mean (+/− SD) percentage of morphologically normal spermatozoa of 87.55 +/− 10.45 in silver fox ejaculates. However, as there are few sperm with abnormal morphology in the ejaculate, and the concept of morphology is closely linked to the dimensions of the sperm, accurate morphometric analysis of spermatozoa takes on greater importance [8,9]. Morphometric evaluation of sperm can be used to predict potential fertility and as a tool in biological studies [10]. Many authors have attempted to find relationships between sperm morphometry and male fertility [11,12,13,14,15]. Studies on human semen have shown that the sperm of infertile men had longer and wider heads [16]. Differences in head size between fertile and infertile males have been found in animals such as stallions, boars and dogs [13,17,18]; animals with smaller sperm head dimensions proved to be more fertile. Furthermore, it has been demonstrated that not only the head size affects fertilization capacity or sperm motility, but also the dimensions of the tail and midpiece [19,20]. 

Using classical staining methods or CASA (Computer Assisted Semen Analysis), it is impossible or very difficult to distinguish the boundaries between the nucleus and the acrosome in the semen of many animal species [10]. Assessment of acrosome morphometry has thus far been carried out on the sperm of just a few mammalian species [10,12,21,22]. This is because most sperm staining techniques also stain the background of the slide, and the sperm head is most often uniformly stained. According to Yániz et al. [10], the acrosome is the most varied, enabling clear differentiation of the sperm of different animal species. Differences in sperm morphology and physiology even between related species have raised many questions about the cause of this diversity. In the present study, we used an experimental method of staining semen specimens with silver nitrate, which allows more precise analysis of individual sperm structures, including the nucleus and acrosome [18,23]. 

The Arctic fox (*Alopex lagopus*) and Red fox (*Vulpes vulpes*) are two separate Canidae species. Their colour variants are the blue fox and silver fox, respectively [24]. The karyotypes of the two species differ in the diploid number of chromosomes and their morphology [25,26]. The genome size differs as well [27]. The karyotype of the blue fox has a variable number of chromosomes (2*n* = 48−50) and no B chromosomes [24,25], while the silver fox karyotype has a constant, diploid number of chromosomes (2*n* = 34) and 0 to 10 additional B chromosomes [24,26,28].

Canine spermatozoa, especially the sperm of farmed foxes, are not often described in studies on reproduction. Precise determination of sperm morphology and morphometry and the establishment of an optimal staining technique for these species are needed in order to obtain objective parameters of sperm quality. The lack of definitive recommendations regarding sperm staining and the lack of reference values for canine morphometric parameters were the inspiration for this study, aimed at providing a detailed comparison of the morphometric dimensions and shape of the sperm of two fox species.

## 2. Materials and Methods

The subject of the study was spermatozoa from one-year-old farmed foxes of two species: silver fox (*Vulpes vulpes*) and blue fox (*Alopex lagopus*). Semen collected from 10 silver foxes and 10 blue foxes was used for the study. Semen was obtained manually during insemination procedures conducted on the farm. Directly after collection, macroscopic and microscopic evaluation of the semen was performed. Sperm motility was evaluated subjectively on a slide placed on a BIOVAL microscope (BIOVAL, Valencia, Spain) stage heated to 37 °C. Semen containing at least 75% spermatozoa with progressive motility was used for further analysis. 

Smears on microscope slides were made from semen diluted with MIII extender (Minitub, Tiefenbach, Germany) to a final concentration of 20 × 10^3^ and dried at room temperature. The specimens were stained with silver nitrate using a modified protocol developed by Andraszek and Smalec [23], based on the basic technique proposed by Howell and Black [29]. A 50% AgNO_3_ solution and a gelatine colloid solution were applied to the specimens in a 1:2 ratio. The smears were covered with a cover slip and incubated for 15–20 min at 60 °C in saturated humidity. When the samples took on a brown colour, the chemical reaction was terminated by washing the specimen several times with distilled water. The specimens were dried at room temperature without access to light. 

Morphometric measurements of the sperm head were performed using the Multiscan image analysis system (Computer Scanning Systems, Warsaw, Poland) connected to an Olympus BX50 (Olympus, Tokyo, Japan) light microscope at 1000× magnification (100× oil immersion objective) and a Jenoptik ProgRes camera (Jenoptik, Jena, Germany). The images were stored in computer memory. Each spermatozoon was measured manually using measurement software coupled with the Multiscan system (Computer Scanning Systems).

Measurements were performed of the length, width, perimeter and area of the head; the area of the acrosome and its coverage (percentage share of the area of the head); the length of the midpiece and its coverage (percentage share of the length of the tail); the length of the tail, and the length of the end piece of the tail. Four morphometric parameters of the head (length, width, perimeter and area) were used to determine four head shape indices: ellipticity, elongation, roughness, and regularity (see Table 1 for formulas). These parameters more precisely characterize the shape of the sperm head. From each fox, 100 morphologically normal spermatozoa from each ejaculate were evaluated. In total, 2000 sperm cells were evaluated (100 cells × 10 individuals × 2 species).

The effect of the species on the morphometric parameters of sperm and the shape indices of the sperm heads was evaluated by one-way analysis of variance using the following mathematical model:

Yij = µ + ai + eij.(1)

where:

Yij—value of feature
µ—mean for population
ai—effect of ith level of factor (species)
eij—sampling error

The significance of differences between groups was verified by Tukey’s test at *p* ≤ 0.05.

Data were analysed by ANOVA using STATISTICA PL 10.0 software (STATISTICA version 10.0, StatSoft Inc., Krakow, Poland).

All experiments were conducted in accordance with Directive EU 86/609/EEC for animal experiments with the approval of the First Local Ethics Committee on Animal Testing at the Jagiellonian University in Kraków. 

## 3. Results

Table 2 presents the morphometric data for the sperm of the two fox species. The data show differences in individual dimensions of the sperm head. Larger sperm head dimensions were observed in the semen of blue foxes. The sperm heads of the blue fox were, on average, nearly 0.4 µm longer than those of the silver foxes (*p* ≤ 0.05). The sperm heads of blue foxes were also 0.33 µm wider on average than those of silver foxes (*p* ≤ 0.05). In addition, they had a larger perimeter and surface area than the sperm heads of silver foxes, by 0.74 µm and 0.83 µm^2^, respectively. The differences were confirmed statistically in both cases (*p* ≤ 0.05). In contrast to the sperm head dimensions, no statistical differences were confirmed in the dimensions of the sperm midpiece or tail between blue and silver foxes. However, it is worth noting that the length of the tail end piece visible in the stained specimen differed significantly in the two species (*p* ≤ 0.05). Blue fox sperm were also longer than silver fox sperm, probably due to the greater length of the sperm heads in blue foxes. The differences were confirmed statistically (*p* ≤ 0.05).

Table 3 presents parameters taking into account the standard dimensions of sperm heads (length, width, perimeter, and surface area) according to Tygerberg strict criteria, which enable a more precise analysis of the shape of the sperm head. In the case of ellipticity, which indicates the degree to which sperm heads are oval, narrow or conical, and elongation, indicating rounding of the sperm head (the closer the value is to 0, the rounder the heads are), no significant differences were found between the sperm heads in the two species. In the case of roughness, a higher value was observed in the sperm of the silver foxes than in the blue foxes (*p* ≤ 0.05). Regularity indicates symmetry of the sperm head and the degree to which it is pyriform (pear-shaped). The higher the regularity value, the more symmetrical the sperm head is. In the present study, the higher regularity value of the sperm heads of blue foxes indicates that they were more symmetrical. This index was 0.1 higher and differed significantly from the regularity index for the sperm heads of silver foxes (*p* ≤ 0.05).

**Figure 1:**
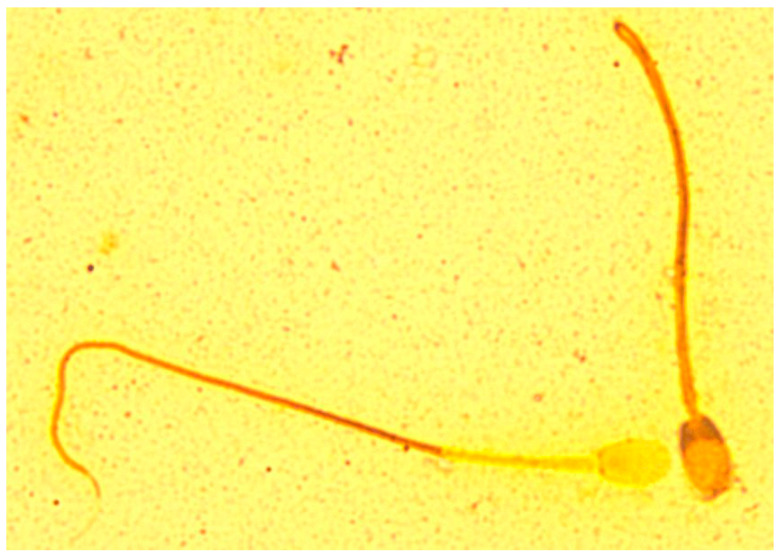
Spermatozoa of blue fox—silver nitrate staining revealing individual sperm structures.

**Figure 2:**
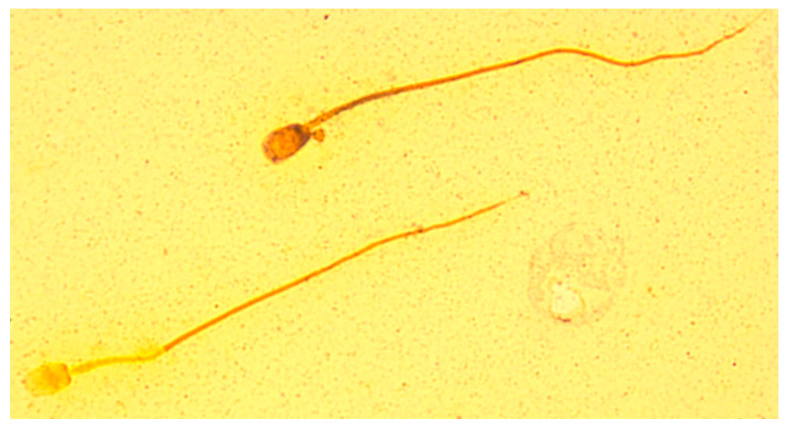
Spermatozoa of silver fox—silver nitrate staining revealing individual sperm structures.

The staining technique used resulted in differences in the staining of individual sperm structures in foxes of both species (Figure 1 and Figure 2). The sperm head and midpiece were negatively stained, revealing individual sperm structures. In the ‘light’ spermatozoa, the acrosome of the head was very light, not much darker than the background of the slide. The equatorial and the post-acrosomal (distal cap) segments were darker than the acrosome. The dark-coloured basal plate of the sperm head was clearly identifiable. The midpiece was light and clearly distinguishable from the rest of the tail. On the border of the midpiece, in its distal part, the distal ring could be identified. The remainder of the tail, including the end piece, was darkly stained and clearly distinct from the background of the slide. In the ‘dark’ spermatozoa, the acrosomal part of the head was clearly distinguishable from the background of the slide. The acrosome coverage could be precisely determined. The equatorial segment of the head formed a clear boundary between the acrosomal and post-acrosomal parts. Due to the dark colouring of the distal cap, it was impossible to identify the basal plate of the sperm head. The midpiece was dark and not easily distinguishable from the rest of the tail. The distal ring was not visible. The remainder of the tail, including the end piece, was dark, and clearly distinguishable from the background of the slide.

## 4. Discussion

The purpose of the study was a detailed comparison of the values of morphometric parameters of the sperm of two fox species blue fox (*Alopex lagopus*) and silver fox (*Vulpes vulpes*). The lack of definitive recommendations regarding sperm staining and the lack of reference morphometric values for canines prompted us to use staining to clearly identify the individual elements of the sperm structure [30]. The staining technique should interfere as little as possible with the cell structure, and, at the same time, should reveal as many details of its structure is possible. The use of sperm staining based on silver nitrate enabled precise measurements of both fox species. Silver nitrate clearly identified the sperm head, differentiating the acrosomal and post-acrosomal parts, and the tail, with a distinctly visible midpiece and end piece. Silver nitrate staining coloured the acrosome less intensively than the post-acrosomal part, and the midpiece was darker than the rest of the tail. This staining enabled precise measurements of individual sperm structures. Silver nitrate is an alkaline dye used to identify acidic chromatin proteins. It differentiates the sperm head more precisely than staining with eosin–gentian violet, which is routinely used for sperm evaluation [30]. This is probably due to the different composition of the cell membrane in the acrosomal and post-acrosomal parts of the sperm [31,32] and the presence in the post-acrosomal part of acidic proteins and nucleoli, which react positively with silver salts [23]. In addition, silver nitrate staining is able to distinguish sperm structures between different species [18,30,33,34,35,36], birds [37], and insects [38], with precise measurements of the sperm structures made in each case. Silver nitrate staining can be used to observe differences in sperm structure between different species [23,33]. Silver nitrate staining standards reveal differences specific to a given species and variety, in both the head of the sperm and the tail [18,30,33,34,35,36]. 

Given that not all laboratories (operating for clinical or breeding purposes) have expensive equipment, the method proposed here could be widely used to address these limitations, as it requires only simple microscopes with measuring software. The high quality of the microscope image, the contrast of the sperm and background, and the ability to identify different parts of the cell make this a very reliable staining method for sperm analysis, which can be used independently or as an auxiliary technique in semen evaluation. 

The sperm measurements made in this study included the surface area, perimeter, length, and width of the head; the acrosome surface area and coverage; the midpiece length and coverage; and the length of the end piece, entire tail, and entire sperm. Species differences in sperm dimensions were noted. The sperm of blue fox were generally larger than those of silver fox; only the acrosome coverage was greater in silver fox. The differences were statistically significant in the case of the area, perimeter, length, and width of the head, surface area of the acrosome, length of the end piece, and total sperm length. The larger size of the blue fox sperm head may be due to differences in the genome size of the two species. The genomes of blue fox and silver fox differ in their diploid number. The blue fox karyotype has a variable number of chromosomes: 2*n* = 48, 2*n* = 49, or 2*n* = 50 [24,25]. This variation is due to Robertsonian translocation and results in a reduction in the number of chromosomes from 2*n* = 50 to 2*n* = 49 or 2*n* = 48 [25,39]. The karyotype of silver fox consists of 34 chromosomes and a variable number of supernumerary B chromosomes [24,26]. The number of additional B chromosomes can range from 0 to 10 [24,28]. These differences in genome size may contribute to the larger size of blue fox sperm, whose genome is much larger than that of silver fox. A similar relationship, pertaining to the nucleus of primary spermatocytes, has been found in wild boar and roe deer. The spermatocyte nucleus was found to have a larger area in roe deer (2*n* = 70) than in wild boar (2*n* = 36) [40]. The greater variation in sperm head parameters and shape indices according to Tygerberg strict criteria in silver fox may also be linked to its karyotype. The blue fox has a constant genome size, with the number of chromosomes resulting from their rearrangement; the number of chromosomes changes, but the size of the genome does not. In the silver fox, the genome is more variable, which is due to the varied number of additional B chromosomes in this species.

A single ejaculate will contain sperm with various shapes, mainly of the head, and with different sizes [41,42]. Furthermore, a single ejaculate contains subpopulations of sperm with varying morphometric and kinematic parameters. Such kinematic, head morphometric and kinetic-morphometric subpopulations have been observed in blue fox [6]. This has prompted researchers to attempt to identify and establish normal characteristics of sperm [41,42], and it is especially interesting to compare differences between related species. For this reason, in the present study we used the Tygerberg strict criteria specifying four shape indices characterizing the sperm head: ellipticity, elongation, roughness, and regularity. The ellipticity index differentiates thin and conical sperm heads. A higher value indicates a thinner sperm head, while a lower value describes a more conical head. Elongation specifies the degree of rounding of the head. If the value is zero, the heads are round, while a higher value indicates a more elongated head. The roughness index identifies rough and amorphous heads, with an uneven cell membrane. The lower the value of this index, the more amorphous the surface of the head is. Regularity defines the symmetry of the sperm head. A low value identifies pear-shaped heads [4,43]. 

Selected morphometric parameters of the sperm of blue fox were also studied by Soler et al. [44]. The authors focused only on the sperm head, but did not analyse the acrosome area or coverage. In that study, the heads of blue fox sperm had smaller dimensions than in our study, and their shape differed as well. In the present study, the heads of sperm in the semen of blue fox were more rounded and their surface was less rough than in the case of sperm from the same species in the study by Soler et al. [6]. The differences in morphometric parameters are likely due to the staining techniques used, Soler et al. [6]. 

Different staining techniques use a wide variety of chemical reagents. In many cases, fixation of the semen on the microscope slide is itself enough to alter the structure of the spermatozoon. Alcohol in various concentrations is commonly used as a fixative of the suspension or smear on the slide. It can cause dehydration and thus shrink the sperm head. Pre-incubation of slides in physiological solution can have a hypotonic effect and cause the sperm head to swell. Reagents and water, by penetrating the sperm membrane to achieve osmotic balance, can cause the cell to swell or shrink [4], hence the differences in the dimensions of sperm of the same species when different staining techniques are used and the need for standardization and optimization of staining techniques for a given species [30]. This is confirmed by the results of the present study. 

Our analysis concerned only morphologically normal sperm to provide a preliminary presentation of the sperm morphometry of two fox species and a precise characterization of the shape of the sperm head. Reference values for the size and shape of sperm make it possible to identify damaged and morphologically abnormal sperm. In the case of animals, including foxes, there are no reference values for the size and shape of sperm. There are also no guidelines regarding staining techniques; a technique that works well in the case of one species may be completely useless in the case of another [36]. Size and shape references and recommendations regarding staining techniques exist only for humans [45]. For animals, there are only guidelines for sperm defects and forms considered morphologically abnormal. A spermiogram has been established for bull semen. This system of classification is also often used to assess boar sperm. A slightly different classification of sperm abnormalities has been developed for stallion semen. The most important sperm defects have also been defined for poultry sperm [36]. However, there are no recommendations regarding staining techniques for individual species.

Thurston et al. [46] conclude that the shape of the sperm head is genetically determined. It is believed to depend on certain factors that may arise during spermatogenesis. Morphologically different gametes then arise, and the genetic factor significantly affects the structure and size of the cell [8,14,46,47]. Both sperm length and velocity are inherited traits [8,48]. Differences in sperm head dimensions can also be determined by the structure and arrangement of the microfibers present in the sperm head. The cytoskeleton of the sperm head consists of nuclear proteins and a nuclear envelope, which are partially responsible for giving shape to the nucleus [49].

Head shape is not a parameter that unambiguously indicates defective sperm. Bulls with a high percentage of sperm with seemingly abnormal conical heads proved to be more fertile than bulls with sperm assessed as normal [50]. Hence, optimal sperm dimensions for specific animal species should be sought and systematized. As shown in the present study, while both species of foxes belong to the family Canidae, there are differences in the size and shape of their sperm.

The size and shape of the sperm cell, as a species-specific trait, can be treated as a biomarker in identifying a species or even a breed. According to Soler et al. [51], the most important parameters of the sperm head, showing the greatest variability enabling identification of dog breeds, are length, ellipticity and elongation. In dogs, genetic isolation between certain breeds must have been sufficient to cause divergence in allele frequency [52]. Research by Soler et al. [53] on camelids of South America found significant differences in sperm head morphometry, which indicates a possible process of gamete isolation. This could apply to the findings of the present study, in which both fox species belong to the family Canidae, and the differences in sperm morphometry are significant and enable species identification. Van der Horst [54] showed that the sperm of most domestic mammals have large elongated heads, while the sperm heads of the African elephant and white rhinoceros are considerably smaller. Rodents show the highest interspecies variation among all mammals [55]. Studies on the semen of related species show that these traits may be useful in making taxonomic distinctions, and in combination with biogeographical, morphological, chromosomal or genetic data can be used to infer phylogenetic relationships. Interesting observations arise from the work of Ding et al. [56], who found that rodents whose sperm heads have an apical hook, which largely contains acrosomal material, have longer sperm tails, while rodent species with oval heads have shorter tails. Evaluation of the sperm midpiece and tail makes it possible to analyse more intricate morphometric subpopulations. Rossi et al. [57] speculate that both morphology and sperm dimensions likely have a strong phylogenetic signal. Sperm morphology should also be considered in conjunction with biogeographic, chromosomal and genetic data, so that comprehensive evolutionary analyses of related species can be performed. 

## 5. Conclusions

The study showed that the silver nitrate staining technique can be effectively used for analysis of fox semen. In practical terms, this is a very quick and easy method that does not require specialized equipment, and can be used in any condition in semen assessment laboratories. The differences in the size and shape of sperm can be used to establish reference patterns for fox sperm enabling more accurate species identification. The present study forms a good conceptual basis for further analysis of canine semen and that of other domestic animal species.

## Figures and Tables

**Table 1:** Formulas used to calculate morphometric measurements of the sperm head.

Parameter	Formula
Head length (µm)	L
Head width (µm)	W
Head perimeter (µm)	P
Head area (µm^2^)	A
Head ellipticity	L/W
Head elongation	(L − W)/(L + W)
Head roughness	4π(A/P^2^)
Head regularity	π(L × W/4 × A)

**Table 2:** Morphometric parameters of fox sperm measured manually with Multiscan software. Different superscript letters designate significant differences between means within rows at *p* ≤ 0.05 (mean ± standard deviation).

Morphometric Parameter	Blue Fox	Silver Fox
Head length (µm)	6.72 ^a^ ± 0.47	6.33 ^b^ ± 0.47
Head width (µm)	4.54 ^a^ ± 0.40	4.21 ^b^ ± 0.44
Head perimeter (µm)	18.11 ^a^ ± 0.74	17.37 ^b^ ± 0.87
Head area (µm^2^)	21.94 ^a^ ± 0.91	21.11 ^b^ ± 1.16
Acrosome area (µm^2^)	11.50 ^a^ ± 0.78	10.92 ^b^ ± 0.77
Midpiece length (µm)	12.85 ^a^ ± 0.40	12.79 ^a^ ± 0.43
Tail end piece length (µm)	3.44 ^a^ ± 0.28	3.28 ^b^ ± 0.28
Tail length (µm)	65.23 ^a^ ± 1.02	65.09 ^a^ ± 1.10
Acrosome coverage (%)	52.43 ^a^ ± 3.11	52.83 ^a^ ± 3.99
Midpiece coverage (%)	19.71 ^a^ ± 0.59	19.65 ^a^ ± 0.59
Sperm length (µm)	71.95 ^a^ ± 1.11	71.42 ^b^ ± 1.26

^a, b^ values within the same row designated with different letters differ significantly (*p* ≤ 0.05).

**Table 3:** Sperm head morphology in fox sperm according to Tygerberg strict criteria. Different superscript letters designate significant differences between means within rows at *p* ≤ 0.05 (mean ± standard deviation).

Morphometric Parameter	Blue Fox	Silver Fox
Ellipticity	1.49 ^a^ ± 0.15	1.52 ^a^ ± 0.19
Elongation	0.19 ^a^ ± 0.05	0.20 ^a^ ± 0.06
Roughness	0.84 ^a^ ± 0.07	0.88 ^b^ ± 0.10
Regularity	1.09 ^a^ ± 0.11	0.99 ^b^ ± 0.13

^a, b^ values within the same row designated with different letters differ significantly (*p* ≤ 0.05).
